# The role of frailty in advanced HF and cardiac transplantation

**DOI:** 10.3389/fcvm.2023.1082371

**Published:** 2023-04-03

**Authors:** Nicole K. Bart, Alice Powell, Peter S. Macdonald

**Affiliations:** ^1^ Heart Transplant Program, St Vincent’s Hospital, Sydney, NSW, Australia; ^2^ School of Medicine, University of Notre Dame, Sydney, NSW, Australia; ^3^ School of Medicine, University of New South Wales, Sydney, NSW, Australia; ^4^ Infiltrative Cardiomyopathy Laboratory, Victor Chang Cardiac Research Institute, Sydney, NSW, Australia; ^5^ Centre for Healthy Brain Ageing, School of Psychiatry, Faculty of Medicine, University of New South Wales, Sydney, NSW, Australia; ^6^ Department of Neurology, Macquarie University Hospital, Sydney, NSW, Australia

**Keywords:** frailty, heart failure, cardiac disease, ageing, superAging, transplantation, robustness

## Abstract

Frailty is a complex, multi-system condition often associated with multimorbidity. It has become an important prognostic maker across a range of conditions and is particularly relevant in patients with cardiovascular disease. Frailty encompasses a range of domains including, physical, psychological, and social. There are currently a range of validated tools available to measure frailty. It is an especially important measurement in advanced HF, because frailty occurs in up to 50% of HF patients and is potentially reversible with therapies such as mechanical circulatory support and transplantation. Moreover, frailty is dynamic, and therefore serial measurements are important. This review delves into the measurement of frailty, mechanisms, and its role in different cardiovascular cohorts. Understanding frailty will help determine patients that will benefit from therapies, as well as prognosticate outcomes.

## Introduction

Frailty is an age-related clinical syndrome and describes a reduced capacity of multiple organ systems with increased susceptibility to stressors and has become an important prognostic indicator across a range of medical conditions including heart failure (HF). The dual pressures of an increasingly older population and need to meaningfully risk stratify patients with chronic illness has led to an explosion in research (1). There are difficulties with estimating frailty prevalence due to a lack of standardisation of concepts and measures in a continually evolving field (2). However, frailty prevalence increases with age and is higher in women than men (3). Higher rates are also seen in ethnic minority groups and those in lower socioeconomic groups (3). Frailty is associated with increased mortality, hospitalisations, worsening mobility, nursing home admissions and lower quality of life (3–5).

There is a clear association between increased health care utilisation and health care costs with a higher degree of frailty (6, 7). Frailty worldwide is estimated to be as prevalent as 15.7% in those aged 80–84 years to be and 26% in those aged >85 years (8). The World Health Organisation has estimated that by 2050, the world's population of people aged 60 years and above will double. With frailty predisposing to healthcare dependency (9), it is a major contributor to global health burden.

## History of cardiac disease and frailty

Ever since research into the definition and clinical implications of frailty began, there has been research into the associations between cardiovascular disease and frailty and its impact (10, 11). Bidirectional relationships were identified early with the prevalence of HF increasing six to sevenfold with increasing frailty severity (10, 12) and the prevalence of frailty two to threefold higher in older individuals with cardiovascular disease (13). An increasing literature has addressed the relationship between frailty, cardiovascular disease, and HF over the past two decades.

## Association between specific pathologies and frailty

There is a strong evidence base for an association between multimorbidity and frailty with a recent meta-analysis identifying that approximately three-quarters of those with frailty had two or more diseases (14). Specifically, there is robust evidence for a greater prevalence and prognostic impact of frailty in coronary artery disease and revascularisation, HF, aortic stenosis and valve replacement, cancer and chemotherapy or cancer surgery, peripheral vascular disease and surgery, general surgery, chronic kidney disease and dialysis, cirrhosis and liver transplantation and critical illness and intensive care unit admission (2).

The overall prevalence of frailty is higher in females (6, 10) In the HF population, women are more likely to have HF with preserved ejection fraction, be older and have a higher comorbidity burden (12).

Although frailty is more prevalent with increasing chronological age, it also occurs independent of age. Frailty is a clinical proxy for biological age. A key goal of ageing research is to determine what factors narrow the gap between chronological and biological age. Another way of expressing this is for health span (those years of life when individuals are functioning well and chronic disease free) to approximate lifespan (15). It follows that frailty is also more prevalent in chronic illness, particularly in the context of long-term inflammation, immune dysregulation, or immune suppression (16, 17). The frailty tools currently in use are modified from their initial application in a geriatric population, and therefore may not address the diverse array of biopsychosocial factors.

**Figure 1:**
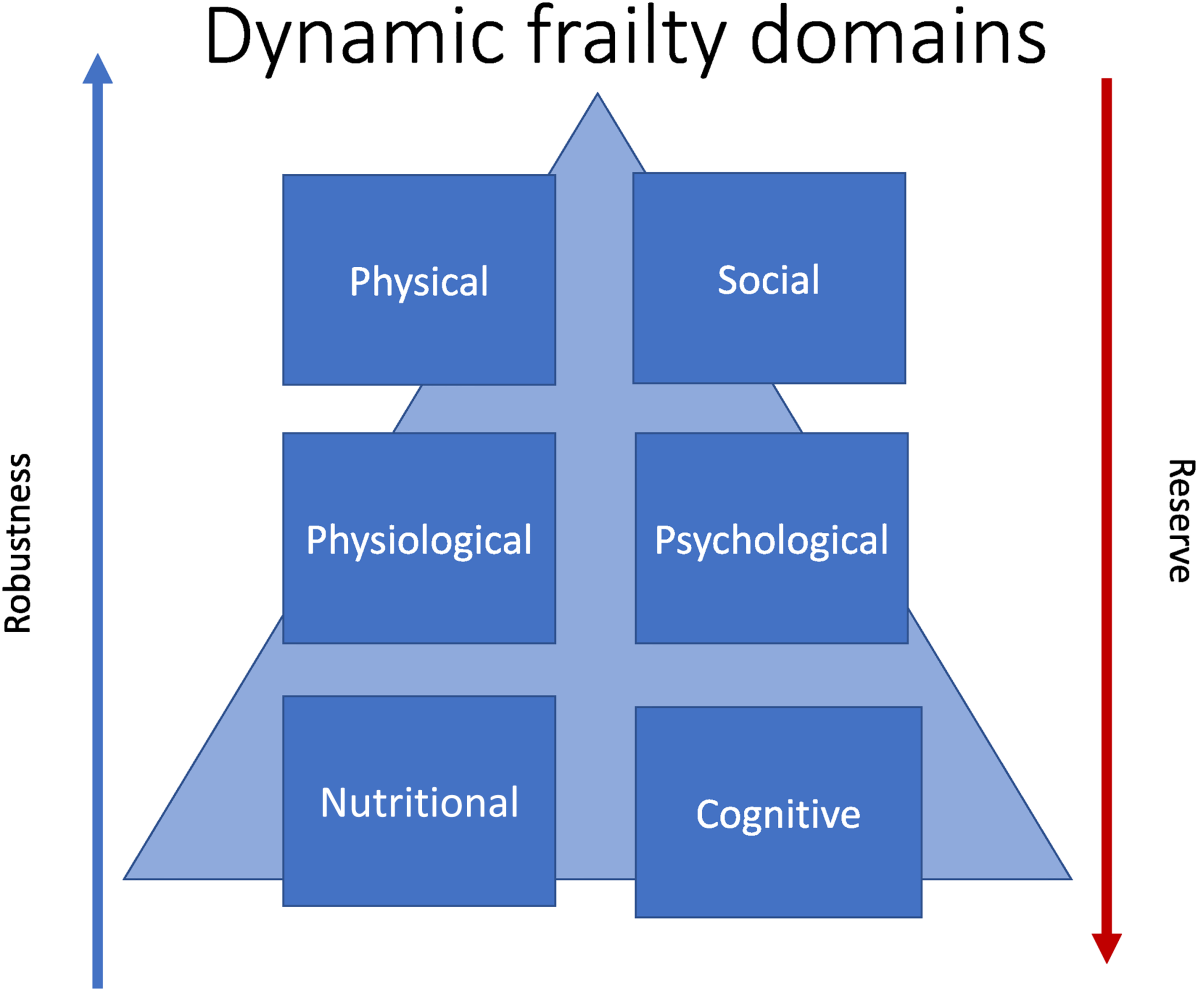
Dynamic frailty domains.

Frailty is multisystem process that is dynamic and potentially preventable. If appropriately recognised and addressed, reversibility of the overall frailty phenotype is also possible. The reverse of frailty is robustness, or a resilience in the face of biopsychosocial stressor (Figure 1). Robustness is related to intrinsic capacity which is defined as all the individual characteristics that contribute to a person's ability to be and to do what they have reason to value (18). This has cognitive, locomotor, vitality, sensory and psychological domains (19).

As will be explored in this review, frailty as a phenotype is a powerful prognostic, but also therapeutic target.

## Definition and communication of frailty

Frailty is defined as reduced reserve in individual with a resultant reduction in ability to tolerate minor or major stressors. The reduced reserve was initially thought to be isolated to the physical domain (10). It is now understood to be the intersection of physical, physiological, immune, cognitive, and social domains. The World Health Organisation on aging broaden the definition to include vulnerability of several organ systems (18). It is important to note, that given the complexity of frailty, there is no universal definition for clinical practice at present (20).

The term “frailty” appeared in the medical literature from the 1950s onwards and was used to describe older people who required health services due to multimorbidity (21). In the 1990s, it was discovered that the incorporation of multiple frailty manifestations better predicted clinical outcomes than any single component alone (22). However, the introduction of the frailty phenotype by Fried and colleagues in 2001 is generally considered the birth of modern frailty research (23). Fried et al. described frailty as a physical phenotype (10) while Mitnitski and Rockwood introduced a frailty index based upon the accumulation of age-related deficits (24). The concept of frailty has evolved over the past two decades with ongoing debate over how it is best defined.

The World Health Organisation (WHO) developed the Integrated Care for Older People (ICOPE) package in 2017 as part of their strategy to implement the Decade of Healthy Ageing Healthy ageing is defined by the WHO as “the process of developing and maintaining the functional ability that enables wellbeing in older age”. The ICOPE program was also designed to maximise intrinsic capacity which is essentially the opposite of frailty and comprises all the mental and physical capacities upon which a person relies to function.

For reference, in this review cognitive frailty refers to cognitive decline in the absence of dementia (25), social frailty refers to loneliness and lack of social networks, and psychological frailty refers to psychological factors that diminish reserve in the event of a stressor, and is sometimes also called “depressive frailty” (9, 26). Finally nutritional frailty (27) is “rapid, unintentional loss of body weight and accompanying disability that often signals the beginning of a terminal decline in an older individual.”

Frailty is a way of communicating with families to help them understand the vulnerabilities of their loved one. A geriatrician popularised the communication of frailty by explaining it as a beautiful “paper boat” to patients and their families (28). If they were to envisage their loved one as a delicate paper boat floating on a pond, then when the pond was quiet and calm, they could sail with no issues. However high winds or storms, which could be a major infection or operation, the boat would no longer be able to sail. This analogy teaches clinicians that they need to assess for, and communicate frailty to patients, especially in the peri-operative or peri-procedural setting. It helps the concept of “shared decision making” and explaining risks (29). Finally, given frailty is dynamic and multi-dimensional, domains such as social networks and nutrition can be supported by families and loved ones.

## Measurement tools

Multiple frailty measurement tools exist in the clinical and research space and although multiple reviews have highlighted the need for a standard measurement, no such tool exists. Moreover, approaches differ between medical specialties. This likely reflects the complexities of frailty and our evolving understanding.

One of the early and best validated tools is the Fried's Frailty Phenotype, also known as the Physical Frailty Phenotype (PFP) (10). It incorporates five domains: unintentional weight lost, self-reported exhaustion, low physical activity level, slow gait speed and weak grip strength with a maximum score of five. A score of 0/5 indicates no frailty (or robustness), 1–2/5 indicates that the patient is prefrail and a score of 3/5 indicates frailty. Limitations of this phenotype include inclusion of grip strength which is not routinely measured in clinical practice and a focus on physical measures alone.

The Clinical Frailty Scale relies on clinician assessment of a patient's level of activity and functional status scored between 1 (very fit) and 9 (terminally ill). The Clinical Frailty Scale (CFS) is very straightforward and widely used in clinical practice and research with an advantage being that scores can be extracted from medical record review. One study evaluated rates of successful resuscitation based on baseline CFS, with no patients with a CFS of 4 or more surviving (30, 31).

Another well validated tool is the deficit accumulation index (DAI), which uses 70 domains with a greater number of health deficits indicating higher frailty (32). Though this score may be more representative of the complexity of frailty domains, it can be clinically cumbersome and time-consuming and is therefore less practical as a repeated measure.

All full discussion of all published frailty tools is beyond the scope of this review. However, Dent et al. have identified 29 different frailty measures and comprehensively reviewed those in common use as well as individual factors such as gait speed (31). Macdonald et al. have also summarised those tools relevant to heart transplantation and failure (33). A new clinical measure called the “Frail Trait Scale” (FTS) is currently being evaluated as part of the FRAILTOOLs project and has been shown to be one of the best tools for predicting a range of adverse outcomes in older people across clinical settings (34).

The American Society of Transplant Surgeons and the American Society of Transplantation, have recommended the modified version of the PFP for measurement of frailty in cardiovascular patients, particularly those referred for transplantation, and that grip strength is a suitable individual measure in non-ambulant patients (35).

## Common pathways for frailty and cardiovascular disease

Moreover, several risk factors for frailty are also risk factors for cardiovascular disease. For example, reduced exercise capacity, and endocrine dysfunction such as insulin resistance increase the rate of cardiovascular disease and frailty (37). Frailty itself may be a risk factor for cardiovascular disease. The inverse is also true, with cardiovascular symptomology and pathophysiology often accelerating the frailty syndrome.

One important common pathway is low-grade inflammation, with both frail patients and those with cardiovascular disease demonstrating higher levels of C-reactive protein (CRP) and IL-6 (38). Inflammation is a hallmark of several types of cardiovascular disease including ischaemic heart disease, and HF (HF). Recent clinical trials have shown that modulating inflammation can prevent cardiovascular diseases (39). For example, the landmark CANTOS study (40) showed than an anti-inflammatory monoclonal antibody could reduce inflammatory biomarkers such as C-reactive protein, and significantly lowered recurrent cardiovascular events. In addition, a recent concept of “inflammageing” (41) has emerged, which is characterized by high levels of inflammatory markers as individuals age. This is strongly correlated with frailty, heart disease and multi-morbidity (42, 43). For example, in the FRAXI study (44), frailty and inflammation were correlated with arterial stiffness. The correlation is explained by a dysfunctional immune state being a common mechanism for each of these conditions (44, 45).

One commonality is nutrition and sarcopenia, which is the involuntary loss of muscle mass and strength. In advanced HF, patients often develop cardiac cachexia, which is a catabolic/anabolic imbalance seen in over 10% of advanced HF patients where nutritional deficiency and sarcopenia is common. This state is also characterized by abnormalities in blood markers such as haemoglobin and albumin (which are also scored on some frailty tools) (13, 46). Interestingly, one of underlying causes of this is chronic inflammation, with these patients having high circulating levels of marker such as IL-6 and TNF-alpha, and high C-reactive protein levels (46). HF patients also show hormonal dysregulation, which can occur before metabolic changes, with impairments in hormones such as leptin, ghrelin, adiponectin (47, 48).

Immune dysregulation has also been implicated in both frailty and cardiovascular disease. Ischaemic heart disease is the leading cause of cardiovascular morbidity worldwide. When myocardial tissue is ischemic both the innate and adaptive immune system is activated (49). With cellular necrosis, and inflammatory cascade is activated. Dysregulation of both the innate and adaptive immune system is also integral to the pathogenesis of frailty (41).

Finally, psychological status affects frailty and cardiovascular outcomes. Comorbid depression and anxiety are common in HF and contributes to frailty. In one prospective study, depression independently increased the risk of HF by 18% over seven years (50). Poor end-organ perfusion, particularly cerebral perfusion is common in the low output state of HF and can be difficult to differentiate from psychomotor motor slowing associated with depression and can also exist concurrently (51). Moreover, HF itself can lead to depression, which then increases the number of HF exacerbations, hospitalisation, and overall risk of death (51).

Frailty is associated with poor outcomes across the spectrum of cardiovascular disease One meta-analysis found that both frailty and pre-frailty was associated with cardiovascular disease, and that frailty was associated with a 3-fold increase in cardiovascular death (52). This is reinforced in other studies which show that frail patients with cardiovascular disease have 2.5–3.5-fold higher mortality than non-frail patients (53). The FRAILTY-AVR (Frailty Assessment Before Cardiac Surgery & Transcatheter Interventions) showed that frailty was as high as 68% in patients undergoing aortic valve replacement, which in turn, increased mortality (13). Across the cardiovascular cohort, frail patients with HF are at greatest risk, with mortality rates as high as 52% (53).

## Overlapping frailty and HF syndromes

It is estimated that over 50% of HF have concomitant frailty (45), which is associated with higher rates of hospitalisation and death (54). The true prevalence of frailty in a HF population is unclear due to the lack of universal definition or frailty tool. The literature is clear however, that HF and frailty are overlapping syndromes. Wang et al., found that in 10 studies involving 3,033 patients, frailty was highly prevalent in patients with HF, with rates of 25.4% to 76% in patients aged >65 years and 70% increased mortality (55). This was replicated in a systematic review of 8 studies and 2,645 patient by Yang et al., where frailty resulted in an 50% increase in hospitalisations and death for HF patients (56).

In the advanced HF spectrum, Jha et al., (54) studied 156 HF patients referred for heart transplantation using the PFP and PFP plus cognition. They found that using both tools, non-frail patients had a higher 12-month survival than frail patients. A further study showed that frailty in an advanced HF population was independent of age, however correlated with NYHA class, body mass index, lower cardiac index, cognitive impairment, and depression. Frailty in this population was an independent predictor of mortality.

There are several studies that show the bi-directional effects of HF and frailty. The mechanisms are several-fold. HF leads to an up regulated neurohormonal state, and chronic inflammation (41). There are higher circulating levels of inflammatory cytokines such as interleukin-1 (IL-1), interleukin-6 (IL-6), C-reactive protein (CRP), and tumour necrosis factor-α (TNF-α), which lead to both tissue depletion and sarcopenia (57). In the HF syndrome, this manifests as dyspnoea, fatigue, and exercise intolerance, which are all contributory to the overall frailty phenotype. Sarcopenia and cachexia are also both present in frailty and HF, and can lead to worse outcomes (58).

There are many common mechanistic pathways that explain the relationship between frailty and HF including effects on the coagulation system, platelets, and endothelium. In in HF, a heightened immune state is present. Risk factors for ischaemic cardiomyopathy, a leading cause of HF including obesity, hypertension, and diabetes and all pro-inflammatory. Moreover, from an early age the process of vascular aging begins leading to a reduction in nitric oxide availably and progressively increasing arterial stiffness (41, 59). This process of “cardiac inflammaging” leads to endothelial dysfunction and myocardial damage. Another key process common to both frailty and HF is mitochondrial dysfunction, which leads to changes in the balance of reactive oxygen species and higher levels of oxidative stress (60). This in turn can lead to platelet and endothelial dysfunction (41).

There are sex differences in the process of cardiac inflammaging. Interestingly, in the same way that frailty is more prevalent in women, there is also a higher prevalence of immune dysfunction, particularly in the form of cardiac inflammaging. The loss of oestrogen that occurs with menopause leads to an up-regulation of pro-inflammatory cytokines, and a decline in anti-inflammatory mechanisms (61). This may explain why women with HF, particularly HF with preserved ejection fraction have worse outcomes than men, although this needs further research.

## Frailty in mechanical circulatory support and transplantation

Frailty is a modifiable risk factor for poor outcomes with advanced HF therapy such as mechanical circulatory support and transplantation. It is an additional diagnostic tool, not currently in the guidelines to help guide the decision making of multi-disciplinary teams. Importantly, it should be measured serially as a patient's journey may change with therapy.

Frailty is a risk factor for post-operative mortality after insertion of left ventricular assist device (LVAD) in both a bridge to transplant and destination therapy cohorts (62).^.^ However, with durable support and treatment of their HF, some patients can recover, and even reverse their frailty. For example, Jha et al., demonstrated that PFP was partly or completely reversible in 12 of 13 patients after LVAD insertion (63).

In contrast, Muthiah et al., (64), showed that patients deemed frail by the PFP had prohibitively poor outcomes post Biventricular assist device (BiVAD) insertion, with mortality rates of *n* = 4/5 in frail patients compared with *n* = 0/6 in non-frail BiVAD supported patients, suggesting that frailty should be a red flag if patients are thought to require BiVAD support during the transplant work-up.

Pre-transplant frailty predicts worse post-transplant outcomes across the range of solid-organ transplants. In a study of 140 heart transplant patients, frailty assessed by the PFP within the 6 months prior to transplant surgery predicted poorer outcomes post-transplant (65). McAdams-DeMarco showed that in renal transplant recipients, PFP score on admission for transplant predicted outcomes (66).

Given the key role that inflammation and immune activation plays in frailty, mapping long term frailty in solid organ recipients would be interesting. Heart transplant recipients have a unique inflammatory predisposition. Regardless of the accuracy of donor-recipient matching, they essentially have a long-term foreign body, and therefore a heightened immune response. Despite improvements in immunosuppression, rejection still occurs in 1/3 recipients and is most prevalence initially post-transplant. The interactions between immunosuppression and frailty are complex, and this is a potential exciting area for research in the future.

It is important to note that frailty is a dynamic process in these complex patients. For example, non-frail patients prior to transplant who have major complications requiring a long hospital admission post-transplant are likely to become frail post-operatively (33).

## Reversal of frailty and future areas of research

One emerging area of future research is the differentiation between fixed vs. dynamic components of frailty. Once this is known, targeted intervention can be applied to optimize reversible elements of the frailty syndrome and increase levels of robustness.

The immune system is central to the process of aging and may play a key role in the dynamic nature of frailty. It may also explain why chronological age may not reflect physiological age. The thymus begins to involute around∼age 15, leading to gradual decline in immune reserve, referred to as immunosenescence (67, 68). A secondary process, inflammaging, may be a potential target for anti-inflammatory therapy to reduce both cardiovascular disease and frailty (41). The more we understand about these dual processes, the more likely this is as a therapeutic target.

Inflammation has become an important therapeutic target in cardiovascular disease and may be a way of also addressing frailty. For example, the CANTOS (69) study showed that monoclonal antibody canakinumab which targets interleukin-1β reduces atherosclerosis. It would be interesting to see what impact such medications could have on frailty in the long-term.

It is important to address and, where possible, reverse cognitive and psychological frailty. Approximately 40 per cent of the population attributable risk of dementia is now considered preventable based on 12 modifiable risk factors including hearing loss, alcohol and smoking, hypertension, physical inactivity and social isolation (70). Strict control of cardiovascular risk factors and prevention of stroke is particularly relevant to people with cardiovascular disease, particularly those with end-stage disease awaiting transplant or supported by an LVAD. For those patients who are hospitalized, prevention of delirium is especially important as this is an independent risk factor for the subsequent development of dementia (71). Other measures recommended in the treatment of mild cognitive impairment such as identification and cessation of medications that may contribute to cognitive impairment, treatment of depression and regular exercise (72) would also be appropriate. Patients with conditions such as depression or dementia treated with anti-depressants can demonstrate neuroplasticity (73). The same may be true for cognitive deficits (independent of reduced cerebral perfusion due to a low output state) in HF patients (74). “Social prescription” as a way of linking older adults with sources of support in the community may combat social frailty although evidence for this intervention is thus far limited (75).

Finally, exercise has been shown to be better than any drug for preventing cardiac disease and maintaining robustness (76). Exercise reduces obesity, insulin resistance, blood pressure and overall cardiovascular disease risk (77). Prehabilitation, defined as the screening for and identification of pre-existing disorder followed by medical optimisation (78), is emerging as a useful tool for assessing patients prior to major cardiac surgery (33). In addition, exercise such as that incorporated in cardiac rehabilitation has been shown to improve prognosis and be excellent secondary prevention once a cardiac event has occurred (79). Determining the appropriate level of exercise intensity for HF patients to maintain safety and maximise benefits is important. The gold standard for such a maximum aerobic exercise intensity prescription is the cardiopulmonary exercise test (CPET) which demonstrates metabolic, respiratory and cardiac responses at anaerobic threshold and respiratory compensation point (80). Where CPET is not available, heart rate during the six minute walk test and step test can guide such prescriptions (81).
